# The Opportunity for Post-Copulatory Sexual Selection in the Ectoparasitic Pea Crab, *Dissodactylus primitivus* (Brachyura: Pinnotheridae)

**DOI:** 10.1371/journal.pone.0145681

**Published:** 2015-12-23

**Authors:** Robert B. Prather, Stephen M. Shuster

**Affiliations:** Department of Biological Sciences, Northern Arizona University, Flagstaff, Arizona, United States of America; Université de Sherbrooke, CANADA

## Abstract

Pea crabs, *Dissodactylus primitivus*, inhabit multiple echinoid (heart urchin) hosts. Male and female crabs move among hosts in search for mates, and both sexes mate multiple times, creating opportunities for post-copulatory sexual selection. For such selection to occur, only a fraction of the males who succeed in mating can also succeed in siring progeny. Jossart et al. 2014 used 4 microsatellite loci to document parentage and mating frequencies of both sexes in *D*. *primitivus*. From these data we identified the mean and variance in female offspring numbers, as well as the proportions of the female population that were gravid and not bearing offspring. We next identified the proportions of the male population who had (1) mated and sired offspring, (2) mated but failed to sire offspring, and (3) failed to mate altogether. We used these results to estimate the opportunity for selection on males and females in terms of mate numbers and offspring numbers, and estimated the sex difference in the opportunity for selection (i.e., the opportunity for sexual selection) using both forms of data. We then partitioned the total variance in male fitness into pre- and post-copulatory components and identified the fraction of the total opportunity for selection occurring in each context. Our results show that the opportunity for selection on each sex was of similar magnitude (0.69–0.98), consistent with this polyandrogynous mating system. We also found that 37% of the total opportunity for sexual selection on males occurred within the context of post-copulatory sexual selection. However, the fraction of the total opportunity for selection that was due to sexual selection, estimated using both mate numbers and offspring numbers, was 9% and 23% respectively. Thus, we further reduced our estimate of the opportunity for post-copulatory sexual selection in *D*. *primitivus* to less than 10% of the total opportunity for selection (0.37 of 0.09 and 0.23 = 0.03 and 0.09). Our results provide the first estimate of the maximum possible strength of post-copulatory sexual selection in crustaceans using this approach.

## Introduction

Crustacean mating systems are highly variable in nature, but the intensity of post-copulatory sexual selection in this taxon is poorly known [1,2]. Jossart et al. [3] addressed this deficiency by investigating the mating system of *Dissodactylus primitivus*, an ectoparasitic pea crab inhabiting echinoids in Discovery Bay, Jamaica. The authors used 4 microsatellite loci to document parentage in clutches of offspring carried by 18 females within the population (*N*_*females*_ collected = 64). In this polyandrogynous species, in which females vary more in their mate numbers than males [3,4], male and female crabs move among hosts in search of mates, and both sexes mate multiple times, creating opportunities for post-copulatory sexual selection. Jossart et al. genotyped a robust sample of each female’s clutch (30–50 embryos) to reveal the average number of males who sired offspring with each female (2.72 ± 1.23, N = 18), as well as for 14 of these females, the identity of all males who deposited sperm into each female’s spermatheca (average number of mates per mating male = 1.25 ± 0.44, N = 32, *N*_*males*_ collected = 55). The largest number of mates per female was 6; the largest number of mates per male was 2. Thus, this unusually detailed study also provided information on the number of males who mated but did not sire offspring.

We reanalyzed the data of Jossart et al. [3] to illustrate a method [2] for documenting the proportion of the total opportunity for selection on males that can be attributed to post-copulatory sexual selection. We also used the data of Jossart et al. [3] to illustrate how the opportunity for selection on males and on females, as well as the sex difference in the opportunity for selection (i.e., the opportunity for sexual selection) can be estimated from such information. Our results illustrate the data and calculations necessary to document the relative strengths of pre- and post-copulatory opportunities for sexual selection and how these results relate to overall selection in both sexes. Although the pattern of paternity revealed in our results do not distinguish sperm competition from cryptic female mate choice [3], they represent the first results documenting the opportunity for of post-copulatory sexual selection in crustaceans using this approach.

We emphasize that by measuring the opportunity for sexual selection within the context of post-copulatory sexual selection, we do not specify which traits might be shaped by such selection [5–7]. However, our approach is in some ways more useful than if we had measured selection directly on traits presumed to be important in this context [8–10]. Here, we measure the variance in relative fitness in terms of mate numbers and offspring numbers, arising from pre- and post-copulatory fertilization success, which we assign explicitly to males and females. Our estimates of the opportunity for selection thus provide an empirical estimate of the maximum possible strength with which selection can act on all traits evolving in this context, or as Crow [5] defined it, “total selection intensity.”

Direct estimates of selection on traits associated with post-copulatory fertilization success will always be less than our estimates for two reasons: (1) the magnitude of the covariance between a particular phenotype (e.g., sperm morphology) and relative fitness, must always lie within the magnitude of the total opportunity for selection [11]; (2) under most circumstances, the covariance between phenotype and fitness for such traits will be less than 1 [8]. Thus, our estimates of the opportunity for selection provide a means for identifying the maximum possible intensity of selection that can arise in the context of post-copulatory sexual selection. Our use of the opportunity for selection also allows us to partition our empirical estimate of the maximum strength of selection into pre-copulatory and post-copulatory components, adding additional precision to our measures [2]. Such information is not available from direct estimates of the covariance between a particular phenotype and fitness [12]. Additional considerations for direct estimates of selection are reviewed elsewhere [5–11].

### Measuring the Mean and Variance in Fitness

The evolutionary consequences of post-copulatory sexual selection (i.e., sperm competition and/or cryptic female mate choice) are presumed to be widespread and significant, particularly in species in which multiple mating by females occurs [4,10,13–17]. Multiple mating by females is thought to create opportunities for post-copulatory sexual selection in males [2], and multiple paternity within female families is now well-documented [2,3,18–21]. However, evidence of multiple insemination alone is not sufficient to allow significant selection to operate in this context.

Shuster and Wade [8, 2] identified two necessary conditions for post-copulatory sexual selection to occur. First, both males and females must have multiple mates. This tendency will be greatest when a positive covariance exists between mate numbers and offspring numbers for each sex. However, if this covariance is positive in only one sex, zero, or negative in sign for one or both sexes, multiple inseminations will occur less frequently, by chance, or not at all [2]. Post-copulatory sexual selection can constitute a significant evolutionary force only if multiple inseminations are common. If multiple inseminations are rare or occur only by chance, they will only constitute a small part of the total distribution of circumstances in which sexual selection can occur. Such conditions will make selection in this context weak. Second, among the males who are successful at mating with multiple females, a fraction of the successfully mating males must sire no offspring at all. The larger this fraction is, the stronger post-copulatory sexual selection can become. Thus a large number of ineffectively mating males is crucial if post-copulatory sexual selection is to have a large effect on the variance in male reproductive success. Recent studies in *Drosophila* [22] have found that only 2% of the variation in male reproductive success is attributable to differential post-copulatory fertilization success.

Wade [7] showed that when males differ in mate numbers, the mean and variance in the number of offspring produced by males (henceforth “male offspring numbers”) can be determined if the mean (*O*_*females*_) and variance (*V*_*Ofemales*_) in offspring numbers for females are known. This is true because a multiplicative relationship exists between male mate numbers and the number of offspring produced by females (henceforth “female offspring numbers”). For example, the average number of offspring per male, *O*_*males*,_ equals the average number of offspring per female, *O*_*females*_, multiplied by the average number of mates per male, *R*, where *R* = *N*_*females*_ / *N*_*males*_. When the sex ratio equals 1, the numbers of males and females in the population are equivalent. Because every offspring has a mother and a father [23], when the total number of offspring is divided by the number of adults of each sex, the average numbers of offspring for each sex are also equivalent. Thus, when *R* = 1, *O*_*males*_ = *O*_*females*_ [8].

A multiplicative relationship also exists between male mate numbers and female offspring numbers for the variance in male fitness. When males are more variable in their mate numbers than females, the variance in male fitness can become much larger than that of females [7–8]. However, this relationship is eroded if females also vary in their mate numbers as in *D*. *primitivus* [3]. In such cases, reduction in the variance in male fitness can be severe [3,8]. Wade [7], see [6,8] also showed that the variance in male fitness, *V*_*Omales*_ divided by the squared average in male fitness, *O* ^2^_*males*_, estimates the opportunity for selection on males, *I*_*males*_. This quantity measures the variance in relative fitness associated with an episode of selection; i.e., the maximum possible strength of selection acting on all traits influenced by a particular selection event.

Wade and Shuster [24] observed that the total variance in male fitness, *V*_*Omales*_, can be expressed in terms of the fractions of mating, *p*_*Smales*_, and non-mating males, *p*_*0males*_ (= 1—*p*_*Smales*_), as well as in terms of the mean and variance in mate numbers, i.e., harem size (*H* and *V*_*H*_ respectively). Because of the multiplicative relationship between mate numbers and offspring numbers described above, the total variance in male fitness can also be expressed in terms of offspring numbers using the mean and variance in offspring numbers for females, *O*_*females*_, *V*_*Ofemales*_ respectively. Thus, the total variance in male fitness expressed in offspring numbers, *V*_*Omales*_, equals the average variance in offspring numbers within the class of successfully mating males, plus the variance in offspring numbers between the classes of mating and non-mating males or,

$$V_{O}{}_{males}=(p_{S}{}_{males})V_{O}{}_{females}+O_{{{}_{f}}_{emales}}^{2}(p_{S}{}_{males})(p_{O}{}_{males}).$$ (1)

As explained elsewhere [8,25] this approach provides a useful means for including the fitness of non-mating males into calculations of fitness for all males within a population, and it facilitates comparison of the average and variance in fitness, expressed in terms of offspring numbers, between males and females.

A similar approach can be used to estimate the average fitness of all males, mating and non-mating, when the average number of mates or offspring produced by mating males, as well as the proportion of mating and non-mating males in a population are known [8,24,25]. In such situations the average fitness of all males is a weighted average of the fraction of the population that consists of non-mating males, *p*_*0males*_, multiplied by their fitness, plus the fraction of the population that consists of mating males, *p*_*Smales*_, multiplied by their fitness. Because the fitness of non-mating males is zero, the first term of this equation disappears and the average number of mates for all males in the population, *M*_*all*_, equals

$$M_{all}\;=\;\left(p_{Smales}\right)\;M_{mating}$$ (2a)

where *M*_*mating*_ equals the average number of mates per mating male. Similarly, if the average number of offspring is known for mating males, the average number offspring for all males, *O*_*males(all)*_, equals,

$$O_{males\left(all\right)}\;=\;\left(p_{Smales}\right)\;O_{males\left(mating\right)}$$ (2b)

where *O*_*males(mating)*_ equals the average number of offspring produced per mating male. As explained elsewhere [8,25] when the average and variance in fitness is estimated only for mating males, the average fitness is over-estimated and the variance in fitness is under-estimated. Thus, when the average and variance in fitness for all males is estimated using the fractions of mating and non-mating individuals in the population, compared to the average and variance in fitness for mating males alone, the average fitness for all males is expected to decrease and the variance in fitness for all males is expected to increase.

Similar relationships exist for the mean and variance in female fitness (e.g., by substituting the average number of mates for all females in the population, *F*_*(all)*_, the fraction of the population that consists of mating females, *p*_*Sfemales*_, and the average number of mates per mating female, *F*_*(mating)*_, into Eq 2a; and by substituting the average number offspring for all females, *O*_*females(all)*_, the fraction of the population that consists of mating females, *p*_*Sfemales*_, and the average number of offspring produced per mating female, *O*_*females(mating)*_ into Eq 2b). Thus, while these parameters of female fitness are usually estimated only for breeding females (e.g., the subset of females (18 of 64) whose families were genotyped in [3]), if the fractions of the breeding and non-breeding female population are known, these parameters can be used to estimate the mean and variance in fitness for all of the females in the population, as we illustrate below.

### Measuring Pre- and Post-Copulatory Sexual Selection

Shuster et al. [2], proposed a method for quantifying the opportunity for post-copulatory sexual selection by identifying three fractions of the male population: (1) males who fail to mate, *p*_*0males*_, (2) males who mate but fail to sire offspring, *p*_*Sm0males*_, and (3) males who mate and sire offspring because they possess competitive or preferred sperm, *p*_*Smales*_. Because all males in the population belong to one of these three groups, the sum of these fractions equals 1 (*p*_*0males*_ + *p*_*Sm0males*_ + *p*_*Smales*_ = 1). Shuster et al. [2] showed that each of these fractions of the male population and thus both pre- and post-mating sexual selection are captured in the equation,

$$V_{O}{}_{males}=(p_{S}{}_{males})V_{O}{}_{females}+O_{{{}_{f}}_{emales}}^{2}(p_{S}{}_{males})(p_{0}{}_{males}+p_{S}{}_{m0males}).$$ (3)

If there are no males in the population who mate and do not sire offspring, that is, *p*_*Sm0males*_ = 0, then Eq 1 = Eq 3. However, if such males exist (i.e, *p*_*Sm0males*_ > 0) and if the expression, *p*_*0males*_ (instead of *p*_*0malses*_ + *p*_*Sm0males*_), is used to estimate the unsuccessful fraction of the male population, then Eq 1 measures the variance in male fitness that is due to all sources of selection EXCEPT that which is caused by differences among males in their ability to sire young after mating. This latter quantity can be considered equal to the variance in male mating success due to premating fitness components, or under these circumstances, when solved, Eq 1 = *V*_*Omales(pre)*_.

As explained in Shuster et al. [2], the total variance in male fitness equals the sum of pre-mating and post-mating fitness components, or, *V*_*Omales*_ = *V*_*Omales(pre)*_ + *V*_*Omales(post)*_. Subtracting *V*_*Omales(pre)*_ from both sides of this equation gives *V*_*Omales(post)*_ = *V*_*Omales*_−*V*_*Omales(pre)*_. Thus, *V*_*Omales(post)*_ is equal to the variance in male fitness that is due to post-mating sexual selection, and this quantity can be estimated explicitly by solving Eqs 1 and 3, and then calculating their difference (e.g., Eq 3 –Eq 1 = *V*_*Omales(post)*_). By dividing this result by the squared average fitness for males, *O*_*males*_^2^, the opportunity for selection among males that is due to post-mating sexual selection, *I*_*males(post)*_ = *I*_*males*_−*I*_*males(pre)*_, is obtained. Moreover, the relative contribution of the opportunity for post-mating sexual selection to other sources of selection can be expressed as the ratio of these parameters. For example, the fraction of the total opportunity for selection on males that is due to post-mating sexual selection can be estimated as *I*_*males(post)*_ / *I*_*males*_. We illustrate this approach below.

## Results

Jossart et al. [3] reported that 55 males and 64 females, 39 of whom were gravid, were collected in their sample. These authors assigned parentage to all of the 758 zygotes in their study using four microsatellite markers, and GERUD 2.0 and COLONY 2.0.2.1 software, which inferred the minimum as well as the most likely number of fathers, respectively. Only two of the 73 adult genotypes in their study were similar, leading to a probability of parentage exclusion with one known parent of over 99%. From these data we estimated the sex ratio as *R* = *N*_*females*_ / *N*_*males*_ = 64/55 = 1.16. We also estimated the fraction of breeding females in this sample, *p*_*Sfemales*_, = 39/64 = 0.61, and the fraction of non-breeding females as, *p*_*0females*_ = 1 –*p*_*Sfemales*_ = 0.39 (S1 Table, rows 1–5).

After genotyping all males and the sample progeny, the authors reported that 32 males sired progeny, with 8 males contributing to 2 clutches. An additional 9 males were identified as having mated with females via spermatheca analysis, but were not represented among the fathers identified within clutches. Thus, of the 55 collected males, a total of 41 males (= 32 + 9) successfully mated, but only 32 of these mating males successfully sired progeny. Following [2], we considered males to be comprised of three classes; (1) males who failed to mate altogether, *p*_*0males*_, (2) males who mated successfully but failed to sire offspring, *p*_*Sm0males*_, and (3) males who mated successfully and sired offspring, *p*_*Smales*_.

We estimated the fraction of successfully mating males as *p*_*Smales*_ + *p*_*Sm0males*_ = 32/55 + 9/55 = 41/55 = 0.745; the fraction of unsuccessfully mating males equaled *p*_*0males*_ = (1 –*p*_*Smales*_ + *p*_*Sm0males*_) = 1–0.745 = (55–41)/55 = 0.255. Of the successfully mating males, the fraction who mated but failed to sire offspring equaled *p*_*Sm0males*_ = (41–32)/55 = 0.164. Thus, the fraction of males who mated and sired offspring, *p*_*Smales*_, equaled 32/55 = 0.582, and the fraction of males who failed to sire offspring regardless of their mating status equaled (1—*p*_*Smales*_) = 0.418. Overall, the three male fractions summed to 1, or *p*_*Smales*_ + *p*_*Sm0males*_ + *p*_*0males*_ = 0.582 + 0.164 + 0.255 = 1.00 (S1 Table, rows 6–9).

Jossart et al. [3] reported the mean and variance in female offspring numbers, as 203 and 1,156 (= 34^2^; N = 9), respectively. We estimated the average number of offspring for males as *O*_*males*_ = *RO*_*females*_ = (1.16)(203) = 236.2, illustrating that male and female fitness are quantitatively linked through the sex ratio [2]. As in Eq 3, we estimated the total variance in offspring numbers for mating and non-mating males as *V*_*Omales*_ = *p*_*Smales*_ *V*_*Ofemales*_ + *O* ^*2*^_*females*_ (*p*_*Smales*_) (*p*_*0males*_ + *p*_*Sm0males*_). Using the above values, we estimated the total variance in male fitness in terms of offspring numbers, *V*_*Omales*_, as 672.58 + 10,026.39 = 10,698.97 (S1 Table, rows 10–13).

We next used Eq 1 to estimate *V*_*Omales(pre)*_, wherein we used only *p*_*0males*_ [instead of (*p*_*0males*_ + *p*_*Sm0males*_)] to estimate the unsuccessful fraction of the male population. This expression provided the variance in male fitness due to all sources of selection EXCEPT that which is caused by differences among males in their ability to sire young after mating, i.e., the variance in male mating success due to premating fitness components. Using the above values we estimated *V*_*Omales(pre)*_, as 672.58 + 6,103.02 = 6,775.60 (S1 Table, row 14).

By subtracting *V*_*Omales(pre)*_ from *V*_*Omales*_ we obtained, *V*_*Omales*_−*V*_*Omales(pre)*_ = *V*_*Omales(post)*_ wherein *V*_*Omales(post)*_ equaled the variance in male fitness due to the effects of post-mating sexual selection. Using the above values, we estimated *V*_*Omales(post)*_ as 10,698.97–6775.60 = 3,923.37 (S1 Table, row 15).

By dividing the above result by the squared average fitness for males, *O*_*males*_^2^ (= (*RO*_*females*_)^2^ = [(1.16)(203)]^2^ = 55,799.0), we obtained the opportunity for selection on males that was due to post-mating sexual selection, *I*_*males(post)*_ = *I*_*males(total)*_—*I*_*males(pre)*_. Using the above values, *I*_*males(post)*_ = 0.070, *I*_*males(total)*_ = 0.192, and *I*_*males(pre)*_ = 0.121. We found that the relative contribution of post-copulatory sexual selection (i.e., sperm competition or cryptic female choice) to other sources of selection, *I*_*males(post)*_ / *I*_*males(total)*_ was 0.367 or about 37%. (S1 Table, rows 16–19).

To place this result within the context of the *D*. *primitivus* mating system, we next estimated the opportunity for selection on females, *I*_*females*_, as well as the sex difference in the opportunity for selection, *I*_*mates*_, i.e., the opportunity for sexual selection [7,8]. Given the data available in Jossart et al. [3], we used two different data sets; (1) the mean and variance in mate numbers for males and females, and (2) the mean and variance in offspring numbers for males and females.

Jossart et al. [3] reported the mean and variance in mate numbers for the 32 males that sired offspring as 1.25 and 0.188 (= 0.44^2^) respectively. For the 18 genotyped females and their families, the reported mean and variance in females mate numbers were 2.72 and 1.234 (= 1.53^2^) respectively (S1 Table, rows 20–23). As reported above, using Eq 1 [where *p*_*0males*_ *=* (*p*_*Sm0males*_ + *p*_*0males*_)] the population frequencies of males who sired offspring, *p*_*Smales*_, and who were unsuccessful in siring offspring, *p*_*0males*_ (defined as in Eq 1) were 0.582 and 0.418 respectively (*N*_*males*_ = 55). The population frequencies of females who were gravid, *p*_*Sfemales*_, and non-gravid, *p*_*0females*_, were 0.609 and 0.391, respectively (*N*_*females*_ = 64).

Following Eq 2a, we estimated the average fitness for all males in terms of mate numbers, *M*_*(all)*_ as (*p*_*Smales*_)(*M*_*(mating)*_) = (0.582)(1.25) = 0.727. Following Eq 1, we estimated the variance in fitness for all males as (*p*_*Smales*_) *V*_*M(mating)*_ + *M* ^2^_*(mating)*_ (*p*_*Smales*_)(*p*_*0males*_) = (0.582)(0.188)+(1.25)^2^(0.582)(0.418) = 0.489. Following [8] we estimated the opportunity for selection on males, in terms of mate numbers, *I*_*males(mates)*_ = *V*_*M(all)*_/*M* ^2^_(all)_ = (0.489)/(0.727)^2^ = 0.925 (S1 Table, rows 24–26).

Following Eq 2a, we estimated the average fitness for all females in terms of mate numbers, *F*_*(all)*_ as (*p*_*Sfemales*_)(*F*_*(mating)*_) = (0.609)(2.72) = 1.659. Following Eq 1, we estimated the variance in fitness for all females as (*p*_*Sfemales*_) *V*_*F(mating)*_ + *F* ^2^_*(mating)*_ (*p*_*Sfemales*_)(*p*_*0females*_) = (0.609)(1.534)+(2.72)^2^(0.609)(0.391) = 2.699. Following [8] we estimated the opportunity for selection on females, in terms of mate numbers, *I*_*females(mates)*_ = *V*_*F(all)*_/*F* ^2^_*(all)*_ = (2.699)/(1.659)^2^ = 0.981(S1 Table, rows 27–29).

The sex difference in the opportunity for selection in terms of mate numbers equaled *I*_*males(mates)*_−*I*_*females(mates)*_ = (0.925)-(0.981) = -0.056. However, because the sex ratio in this study was somewhat female-biased, we accounted for this effect using Eq 1.24b, p. 29 from [8] wherein *I*_*males(mates)*_ *−I*_*females(mates)*_ = (1/*R*−1) *I*_*females(mates)*_ + *I*_*mates*_. Using the above values, solving for *I*_*mates*_ provided an estimate of the opportunity for sexual selection, adjusted for the biased sex ratio. Here *I*_*mates(adj)*_ = 0.082. The fraction of the total opportunity for selection on males due to sexual selection, in terms of mate numbers, *I*_*mates(adj)*_/*I*_*males(mates)*_ = (0.082)/(0.925) = 0.089, or about 9% (S1 Table, rows 30–31).

We had already estimated the total variance in male fitness for all males in terms of offspring numbers (*V*_*Omales*_ = 10,698.97). Using Eq 2b, we estimated the average fitness for all males as (*p*_*Smales*_)(*O*_*males(mating)*_) = (*p*_*Smales*_)(*O*_*females*_) = (0.582)(203) = 118.1. The opportunity for selection on males in terms of offspring numbers, *I*_*males(offspring)*_ = *V*_*Omales(all)*_/*O* ^2^_*males(all)*_ = (10,698.97)/(118.1)^2^ = 0.767 (S1 Table, rows 32–34).

Following Eq 2b, we estimated the average fitness for all females in terms of offspring numbers as (*p*_*Sfemales*_)(*O*_*females(mating)*_) = (0.609)(203) = 123.7, and following Eq 1, we estimated the variance in fitness for all females as (*p*_*Sfemales*_) *V*_*Ofemales(mating)*_ + *O* ^2^_*females(mating)*_ (*p*_*Sfemales*_)(*p*_*0females*_) = (0.609)(1156.0)+(203)^2^(0.609)(0.391) = 10,513.72. The opportunity for selection on females in terms of offspring numbers, *I*_*females(offspring)*_ = *V*_*Ofemales(all)*_/*O* ^2^_*females(all)*_ = (10,513.72)/(123.7)^2^ = 0.687 (S1 Table, rows 35–37).

The sex difference in the opportunity for selection in terms of offspring numbers, equaled *I*_*males(offspring)*_−*I*_*females(offspring)*_ = (0.767)-(0.687) = 0.08. However, again, because the sex ratio in this study was somewhat female-biased, we accounted for this effect using Eq 1.24b, p. 29 from [8] where *I*_*males(offspring)*_ *−I*_*females(offspring)*_ = (1/*R*−1) *I*_*females(offspring)*_ + *I*_mates*_. Using the above values, solving for *I*_mates*_ provided an estimate of the opportunity for sexual selection in terms of offspring numbers, adjusted for the biased sex ratio. Here *I*_mates(adj)*_ = 0.177. The fraction of the total opportunity for selection on males due to sexual selection, in terms of offspring numbers, *I*_mates(adj)*_ /*I*_*males(offspring)*_ = (0.177)/(0.767) = 0.230, or about 23% (S1 Table, rows 38–39).

## Discussion

Our results show how published results can be mined to broaden understanding of how selection in different contexts may shape mating system evolution. We illustrate a simple method [2] for comparing the fitnesses of males and females in natural populations using parentage data and we apply it to estimate the opportunity for post-copulatory sexual selection on males. Fine-grained parentage data like those reported in Jossart et al. [3] are becoming increasingly available, but they must be interpreted with caution because by necessity, they focus only on a small subset of the population; i.e., the males and females in the sampled population who were genotyped and could be assigned offspring. However, using estimates of the mean and variance in fitness from these individuals, either in terms of mate numbers or in terms of offspring numbers, and by combining these results with estimates of the fractions of the population that succeed or fail in producing offspring, a remarkably clear picture of a species mating system and how selection operates within it is obtained.

We used data from Jossart et al. [3] to estimate the relative magnitudes of the opportunity for pre- and post-copulatory sexual selection in a natural population of pea crabs, and provide, to our knowledge, the first estimate of the maximum intensity of sperm competition in terms of the opportunity for post-mating sexual selection in crustaceans. We estimated that the fraction of the total opportunity for selection on males that could be attributed to post-copulatory sexual selection (either sperm competition or cryptic female choice) was about 37%. This is a sizable proportion given that other studies have shown only small fractions (< 5%) of total selection explainable in this context (review in [2]).

However, our estimate must be further reduced. Although 37% of the total opportunity for sexual selection in pea crabs is due to post-copulatory processes, the fraction of the total opportunity for selection on males that could be attributed to sexual selection was only 9% if estimated using the variance in mate numbers, and 23% if estimated using offspring numbers. Because 37% of 9% and 23% equals 3% and 9% respectively, our results indicate that the maximum possible intensity of selection that can act on traits associated with post-mating competition in *D*. *primitivus* represents less than 10% of the maximum total intensity of selection that can act on all traits in this species. This result is consistent with recent estimates in other species of the strength of post-copulatory sexual selection [2,22].

**Fig 1:**
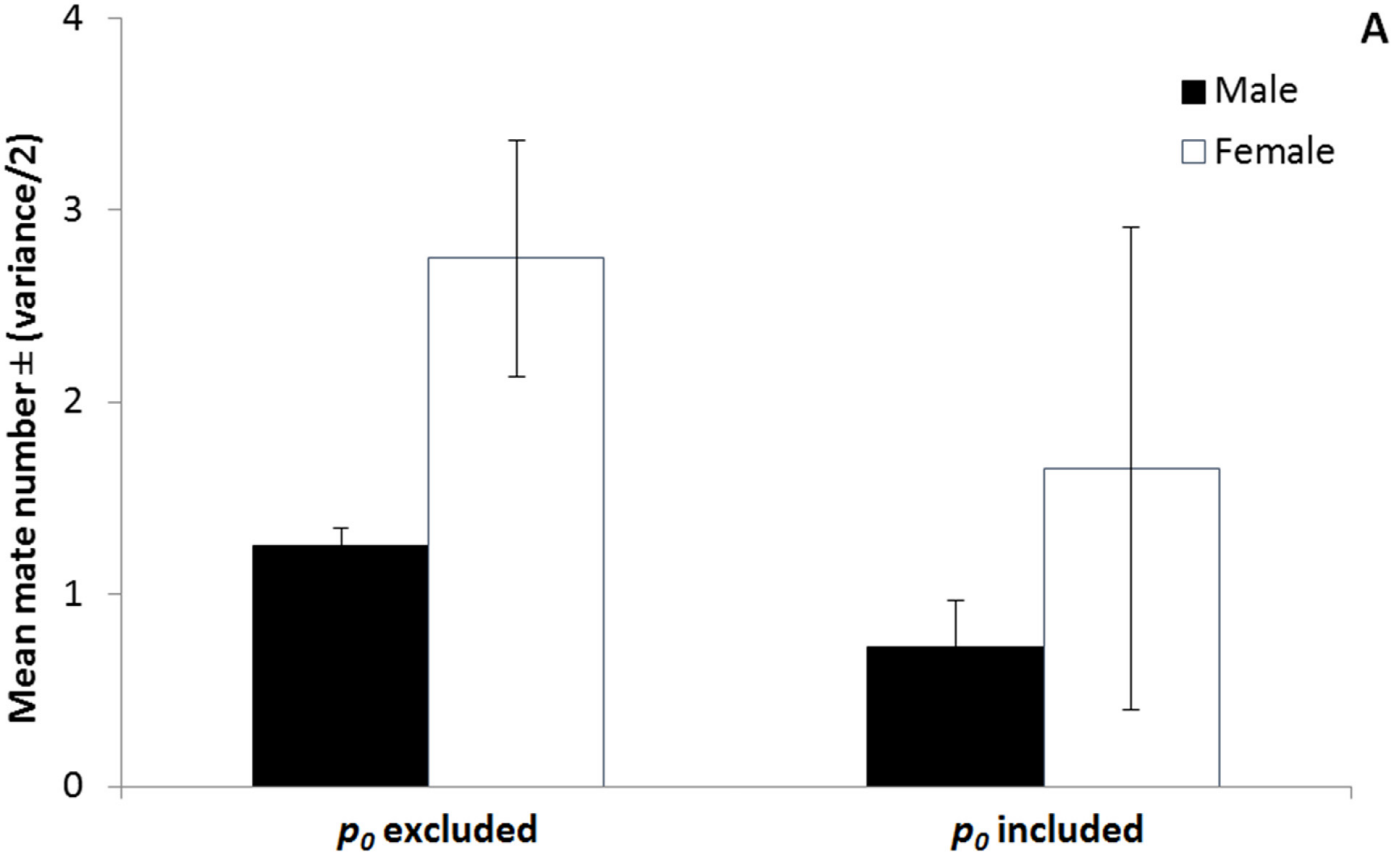
The effect of excluding and including the zero class of individuals (*p*_*0*_). When the mean and variance in fitness are estimated using mate numbers, for males (black bars) and for females (white bars), including the fraction of the population that fails to mate (*p*_*0males*_; *p*_*0females*_) causes the average fitness to decrease and the variance in fitness to increase; error bars represent one half of the variance in fitness.

**Fig 2:**
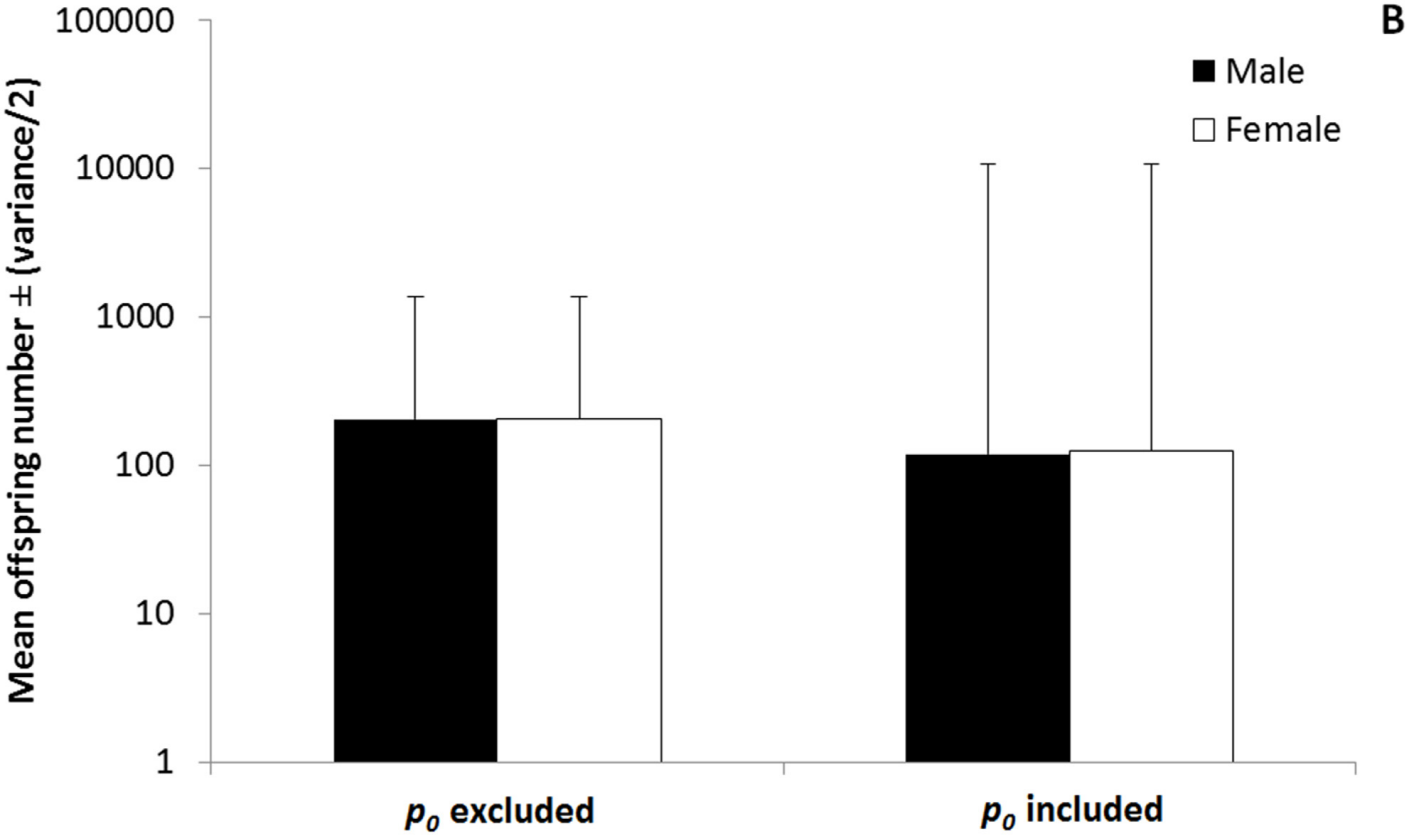
The effect of excluding and including the zero class of individuals (*p*_*0*_). When the mean and variance in fitness are estimated using offspring numbers, for males (black bars) and for females (white bars), including the fraction of the population that fails to produce offspring (*p*_*0males*_; *p*_*0females*_) causes the average fitness to decrease and the variance in fitness to increase; The error bars represent one half of the variance in fitness; only positive values are included; the Y-axis is measured using a logarithmic scale.

Our results showed other important relationships as well. First, we showed how fitness in terms of offspring numbers in one sex is connected to fitness in terms of offspring numbers in the other sex through the sex ratio. Because females were more common than males in this *D*. *primitivus* population, when linked through the sex ratio, the overall average in offspring numbers for males was slightly higher than for females. However, when the fractions of mating and non-mating individuals within each sex were included in our estimates of the mean and variance in fitness for males and females, the larger fraction of non-mating individuals among males than among females reversed this relationship. Specifically, including individuals who were successful and unsuccessful in producing progeny in our estimates of the mean and variance in fitness for both sexes, caused a decrease in the average and an increase in the variance in fitness, for estimates using mate numbers as well as for estimates using offspring numbers (Figs 1–2).

Our estimates of the opportunity for selection in both males and females also allowed us to determine whether a sex difference in the opportunity for selection existed in this species, We found that it did, and despite the fact that in this polyandrogynous species, females were more variable in their mate numbers than males, *I*_*mates*_ was positive in sign, indicating that the opportunity for sexual selection was stronger on males overall than on females. Nevertheless, despite a larger opportunity for selection on males, the fact that both sexes were variable in their mate numbers made the maximum possible net sexual selection on males comparatively weak, a consideration that allowed us to adjust downward our original estimate of the opportunity for post-copulatory selection on males.

Such subtleties are not possible from parentage data alone. We therefore advocate that parentage analyses expand beyond the current practice of reporting only the mean and variance in offspring produced by the males and females that comprise the genotyped sample. We suggest that additional analyses, that include estimates of the fractions of the population that fail to mate, or mate but fail to produce offspring, in addition to those that succeed in producing offspring [1,2,7,8,24,25] are necessary. A clearer picture of mating system evolution is likely to emerge.

## Methods

We used the above statistical approach and the data available in Jossart et al. [3] to identify the fractions of individuals within the *D*. *primitivus* population who were successful and unsuccessful in mating and producing offspring. We quantified the mean and variance in male and female fitness in terms of mate numbers and offspring numbers, and we used this information to illustrate the method of Shuster et al. [2] for estimating the proportion of the total opportunity for selection on males that can be explained by post-copulatory sexual selection. Lastly, we placed these results in the context of the *D*. *primitivus* mating system by comparing the magnitude for selection in this context with the opportunity for selection within each sex, as well as with the magnitude of the sex difference in the opportunity for selection, i.e., sexual selection.

## Supporting Information

S1 Table Mating System Parameters for *Dissodactylus primitivus*. (DOCX)
