# Supplementary material for: The Opportunity for Post-Copulatory Sexual Selection in the Ectoparasitic Pea Crab, Dissodactylus primitivus (Brachyura: Pinnotheridae)
Source: PLoS One. 2015 Dec 23;10(12):e0145681. doi: 10.1371/journal.pone.0145681 (PMC4689395; doi:10.1371/journal.pone.0145681)
Supplement: S1 Table — (DOCX) [file pone.0145681.s001.docx]

| **S1 Table. Mating System Parameters for *Dissodactylus primitivus*** | | | | | |
| --- | --- | --- | --- | --- | --- |
| **Row** | **Parameter** | **Description of Parameter** | **Calculation** | **Value** | **Source** |
| 1 | *N_males_* | Number of males sampled |  | 55 | [3] |
| 2 | *N_females_* | Number of females sampled |  | 64 | [3] |
| 3 | *R* | Sex ratio | 64 / 55 | 1.16 | *N_females_* / *N_males_* |
| 4 | *p_Sfemales_* | Fraction of breeding females | 39 / 64 | 0.61 | 39 / *N_females_* |
| 5 | *p_0females_* | Fraction of non-breeding females | 25 / 64 | 0.39 | 25 / *N_females_* |
| 6 | *p_Smales_* | Fraction of males who sired offspring | 32 / 55 | 0.582 | 32 / *N_males_* |
| 7 | *p_Sm0males_* | Fraction of males who mated, but failed to sire offspring | 9 / 55 | 0.164 | 9 / *N_males_* |
| 8 | *p_0males_* | Fraction of non-mating males | 14 / 55 | 0.255 | 14 / *N_males_* |
| 9 | *1 - p_Smales_* | Fraction of males who failed to sire offspring regardless of their mating status | 1 - 0.582 | 0.418 | *1 - p_Smales_* |
| 10 | *O_females_* | Mean female offspring number^a^ |  | 203 | [3] |
| 11 | *V_Ofemales_* | Variance in female offspring number^a^ | 34^2^ | 1156 | [3] |
| 12 | *O_males_* | Mean male offspring number | (1.16)(203) | 236.2 | (*R*)(*O_females_*) |
| 13 | *V_Omales_* | Total variance in male offspring number | (0.582)(1,156.0) + (203)^2^(0.582)(0.418) | 10,698.97 | Eq. 3 |
| 14 | *V_Omales(pre)_* | Variance in male fitness due to pre-mating processes | (0.582)(1,156.0) + (203)^2^(0.582)(0.255) | 6,775.60 | Eq. 1 |
| 15 | *V_Omales(post)_* | Variance in male fitness due to post-mating processes | 10,698.97 - 6,775.60 | 3,923.37 | *V_Omales_ - V_Omales(pre)_* |
| 16 | *I_males_* | Total opportunity for selection upon males | 10,698.97 / 55,799.0 | 0.192 | (*V_Omales_*) / (*O_males_*)^2^ |
| 17 | *I_males(pre)_* | Opportunity for selection due to pre-mating sexual selection | 6,775.60 / 55,799.0 | 0.121 | (*V_Omales(pre)_*) / (*O_males_*)^2^ |
| 18 | *I_males(post)_* | Opportunity for selection due to post-mating sexual selection | 3,923.37 / 55,799.0 | 0.070 | (*V_Omales(post)_*) / (*O_males_*)^2^ |
| 19 | *I_males(post)_* / *I_males(total)_* | The relative contribution of post-copulatory sexual selection to other sources of selection | 0.070 / 0.192 | 0.367 | [2] |
| 20 | *M_(mating)_* | Average fitness for mating males in terms of mate numbers^b^ |  | 1.25 | [3] |
| 21 | *V_M(mating)_* | Variance in fitness for mating males in terms of mate numbers^b^ | 0.44^2^ | 0.188 | [3] |
| 22 | *F_(mating)_* | Average fitness for mating females in terms of mate numbers^c^ |  | 2.72 | [3] |
| 23 | *V_F(mating)_* | Variance in fitness for mating females in terms of mate numbers^c^ | 1.24^2^ | 1.534 | [3] |
| 24 | *M_(all)_* | Average fitness for all males in terms of mate numbers | (0.582)(1.25) | 0.727 | Eq. 2a |
| 25 | *V_M(all)_* | Variance in fitness for all males in terms of mate numbers | (0.582)(0.188) + (1.25)^2^(0.582)(0.418) | 0.489 | Eq. 1 |
| 26 | *I_males(mates)_* | Opportunity for selection upon males in terms of mate numbers | (0.489) / (0.727)^2^ | 0.925 | [8] |
| 27 | *F_(all)_* | Average fitness for all females in terms of mate numbers | (0.609)(2.72) | 1.659 | Eq. 2a |
| 28 | *V_F(all)_* | Variance in fitness for all females in terms of mate numbers | (0.609)(1.534) + (2.72)^2^(0.609)(0.391) | 2.699 | Eq. 1 |
| 29 | *I_females(mates)_* | Opportunity for selection upon females in terms of mate numbers | (2.699) / (1.659)^2^ | 0.981 | [8] |
| 30 | *I_mates(adj)_* | Opportunity for sexual selection in terms of mate numbers, adjusted for the biased sex ratio | (0.925 - 0.981) - ((1 / 1.16) - 1)(0.981) | 0.082 | Eq. 1.24b from [8, p.29] |
| 31 | *I_mates(adj)_*/*I_males(mates)_* | The fraction of the total opportunity for selection on males in terms of mate numbers that is due to sexual selection | 0.082 / 0.925 | 0.089 | [2] |
| 32 | *O_males(all)_* | Average fitness for all males in terms of offspring numbers | (0.582)(203) | 118.1 | Eq. 2b |
| 33 | *V_Omales_* | Variance in male fitness in terms of offspring numbers | (0.582)(1,156.0) + (203)^2^(0.582)(0.418) | 10,698.97 | Eq. 3 |
| 34 | *I_males(offspring)_* | Opportunity for selection upon males in terms of offspring numbers | (10,698.97) / (118.1)^2^ | 0.767 | (*V_Omales(all)_*) / *(O_males(all)_*)^2^ |
| 35 | *O_females(all)_* | Average fitness for all females in terms of offspring numbers | (0.609)(203) | 123.7 | Eq. 2b |
| 36 | *V_Ofemales_* | Variance in fitness for all females in terms of offspring numbers | (0.609)(1,156.0) + (203)^2^(0.609)(0.391) | 10,513.72 | Eq. 1 |
| 37 | *I_females(offspring)_* | Opportunity for selection upon females in terms of offspring numbers | (10,513.72) / (123.7)^2^ | 0.687 | (*V_Ofemales(all)_*) / (*O_females(all)_*)^2^ |
| 38 | *I_mates(adj)*_* | Opportunity for sexual selection in terms of offspring numbers, adjusted for the biased sex ratio | (0.767 - 0.687) - ((1 / 1.16) - 1)(0.687) | 0.177 | Eq. 1.24b from [8, p.29] |
| 39 | *I_mates(adj)*_* /*I_males_* | The fraction of the total opportunity for selection on males due to sexual selection | 0.177 / 0.767 | 0.23 | [2] |
| ^a^ For 9 of the sampled females, ^b^ For the 32 males that sired offspring, ^c^ For the 18 genotyped females | | | | | |
